# *Candida tropicalis* is the most prevalent yeast species causing candidemia in Algeria: the urgent need for antifungal stewardship and infection control measures

**DOI:** 10.1186/s13756-020-00710-z

**Published:** 2020-04-07

**Authors:** Youcef Megri, Amir Arastehfar, Teun Boekhout, Farnaz Daneshnia, Caroline Hörtnagl, Bettina Sartori, Ahmed Hafez, Weihua Pan, Cornelia Lass-Flörl, Boussad Hamrioui

**Affiliations:** 1 Parasitology and Mycology Department, Mustapha University Hospital, 16000 Algiers, Algeria; 2 Yeast Department, Westerdijk Fungal Biodiversity Institute, Utrecht, The Netherlands; 3 Institute of Biodiversity and Ecosystem Dynamics, University of Amsterdam, Amsterdam, the Netherlands; 4 Institute of Hygiene and Medical Microbiology, Medical University of Innsbruck, Innsbruck, Austria; 5 Biotechvana, 46980 Paterna, Valencia Spain; 6 Shanghai Key Laboratory Molecular Medical Mycology, Shanghai, 200003 China

**Keywords:** Candidemia, Microsatellite typing, Algeria, Antifungal susceptibility testing, MALDI-TOF MS, *ERG11* sequencing, Environmental screening

## Abstract

**Background:**

Despite being associated with a high mortality and economic burden, data regarding candidemia are scant in Algeria. The aim of this study was to unveil the epidemiology of candidemia in Algeria, evaluate the antifungal susceptibility pattern of causative agents and understand the molecular mechanisms of antifungal resistance where applicable. Furthermore, by performing environmental screening and microsatellite typing we sought to identify the source of infection.

**Methods:**

We performed a retrospective epidemiological-based surveillance study and collected available blood yeast isolates recovered from the seven hospitals in Algiers. To identify the source of infection, we performed environmental screening from the hands of healthcare workers (HCWs) and high touch areas. Species identification was performed by API Auxa-Color and MALDI-TOF MS and ITS sequencing was performed for species not reliably identified by MALDI-TOF MS. Antifungal susceptibility testing followed CLSI M27-A3/S4 and included all blood and environmental yeast isolates. *ERG11* sequencing was performed for azole-resistant *Candida* isolates. Microsatellite typing was performed for blood and environmental *Candida* species, where applicable.

**Results:**

*Candida tropicalis* (19/66) was the main cause of candidemia in these seven hospitals, followed by *Candida parapsilosis* (18/66)*, Candida albicans* (18/66), and *Candida glabrata* (7/66). The overall mortality rate was 68.6% (35/51) and was 81.2% for *C. tropicalis*-infected patients (13/16). Fluconazole was the main antifungal drug used (12/51); 41% of the patients (21/51) did not receive any systemic treatment. *Candida parapsilosis* was isolated mainly from the hands of HCWs (7/28), and various yeasts were collected from high-touch areas (11/47), including *Naganishia albida, C. parapsilosis* and *C. glabrata*. Typing data revealed interhospital transmission on two occasions for *C. parapsilosis* and *C. glabrata*, and the same clone of *C. parapsilosis* infected two patients within the same hospital. Resistance was only noted for *C. tropicalis* against azoles (6/19) and fluconazole-resistant *C. tropicalis* isolates (≥8 μg/ml) (6/19) contained a novel P56S (5/6) amino acid substitution and a previously reported one (V234F; 1/6) in Erg11p*.*

**Conclusions:**

Collectively, our data suggest an urgent need for antifungal stewardship and infection control strategies to improve the clinical outcome of Algerian patients with candidemia. The high prevalence of *C. tropicalis* joined by fluconazole-resistance may hamper the therapeutic efficacy of fluconazole, the frontline antifungal drug used in Algeria.

## Introduction

Bloodstream infections caused by *Candida* species, i.e.*,* candidemia, are attributable to the annual high rate of mortality worldwide [1] and significant hospital costs of $1.4 billion in the US each year [2]. The five most prevalent gut mycobiota constituents, i.e.*, Candida albicans, Candida tropicalis, Candida parapsilosis, Candida glabrata,* and *Pichia kudriavzveii* (*C. krusei*) [3] are the major causes of candidemia [4]. Historically, *C. albicans* is known to be the most prevalent cause of candidemia, but the changing landscape of candidemia epidemiology showed that the prevalence of non-*albicans Candida* (NAC) species is increasing [4] and in some cases surpassing that of *C. albicans* [5]. Unfortunately, some of the NAC species, such as *C. glabrata* [6] and *Pichia kudriavzveii* [7], intrinsically have higher minimum inhibitory concentration (MIC) values toward azoles, and *C. glabrata* rapidly acquires resistance to echinocandins [6], the frontline antifungal recommended for the treatment of candidemia [8]. Presently, numerous studies in different countries reported the emergence of *C. parapsilosis* [9] and *C. tropicalis* [10] blood isolates resistant to fluconazole, the frontline antifungal drug used to treat candidemia in developing countries [5, 11]. Most troubling, the emergence of multidrug-resistant strains of *C. glabrata* [6] and, more recently, *C. auris* [12] has led to worrisome therapeutic challenges. Azole resistance mechanisms in *C. albicans, C. parapsilosis*, and *C. tropicalis* is mediated mainly by the occurrence of specific amino acid substitutions in *ERG11*, resulting in reduced affinity of azoles to the drug target, in addition to overexpression of efflux pumps [7].

*Candida* species differ in their mode of transmission in the clinical setting. For instance, *C. albicans* candidemia is acquired mostly endogenously [13], while *C. parapsilosis* is known for being transferred from the hands of healthcare workers (HCWs) resulting in clonal outbreaks in healthcare settings [14]. On the other hand, controversies exist regarding the mode of transmission of *C. tropicalis*, with some believing that it might be horizontally transferred in hospitals [15], while others suggest it is acquired from environmental origins outside of the hospital setting [10]. Regarding *C. glabrata*, although its infection source is generally endogenous, some studies have found horizontal transfer for this species [16]. As a result, resolutive typing techniques, such as microsatellite typing, are of paramount importance to identify the source of infection [14].

Despite compelling evidence about its importance, a comprehensive study of candidemia in Algeria is lacking. Therefore, we conducted the current study to fill this gap. Yeast isolates collected from 2016 to 2019 from seven hospitals in Algiers were identified and subjected to antifungal susceptibility testing (AFST). The contribution of *ERG11* mutations to fluconazole resistance was assessed by *ERG11* sequencing of fluconazole-resistant isolates. Environmental screening followed by microsatellite typing was performed to understand the molecular epidemiology of *C. parapsilosis, C. tropicalis,* and *C. glabrata*.

## Methods

### Settings and study design

This study was approved by the ethical committee of Mustapha Pasha University Hospital. Yeast isolates collected from 2016 to 2019 regardless of age, sex, underlying conditions, and wards were included in this study. Isolates belonged to seven hospitals in Algiers, namely Mustapha Pacha, Beni Messous, Tizi Ouzou, Parnet, and Blida, EPH Médéa, and EPH Zemirli. Blood isolates were obtained from positive blood bottle cultures incubated in Bactec Device (BD BACTEC™ FX Series, Le Pont-de-Claix, France), from which 100 μl was transferred onto Sabouraud chloramphenicol agar (SCA) and chromogenic plates (CandiSelect™ 4, Bio-Rad, Marnes-la-Coquette, France), followed by incubation at 37 °C for 24–48 h.

### Environmental sampling and identification strategy

Environmental sampling was performed using sterile cotton swabs moistened with sterile normal saline. Forty-seven swab samples were taken from high touch spots and reusable devices and 28 from the hands of HCWs. Swab samples were streaked onto two SDA plates, one containing chloramphenicol and one without, and incubated at 37 °C for 48–72 h. Plates without growth of yeasts and those with filamentous fungi were excluded from this study. Yeasts were initially identified by API Auxa-Color (Bio-Rad, Marnes-la-Coquette, France) and further characterized by the MALDI Biotyper system (Bruker Daltoniks, Bremen, Germany) using the full-extraction method [17]. Some rare yeast species belonging to the genera of *Aureobasidium* and *Naganishia* were further confirmed using internal transcribed spacer ribosomal DNA (ITS rDNA) sequencing via the ITS1 and ITS4 primers [18]. DNA samples were extracted using a CTAB-based buffer and following the suggested protocol [17].

### Antifungal susceptibility testing (AFST)

To determine the MIC values of each species, the broth microdilution method using CLSI-M27/A3 was followed [19]. AFST included the following antifungals; fluconazole (FLZ) (Sigma-Aldrich, St. Louis, MO, USA), voriconazole (VRZ) (Sigma-Aldrich, St. Louis, MO, USA), itraconazole (ITZ) (Sigma-Aldrich, St. Louis, MO, USA) anidulafungin (AND) (Pfizer, NY, USA), micafungin (MFG) (Astellas, Munich, Germany), and amphotericin B (AMB) (Sigma-Aldrich, St. Louis, MO, USA). MIC values were visually read after 24 h of incubation at 35 °C, and *Pichia kudriavzveii* (ATCC 6258) and *C. parapsilosis* (ATCC 22019) were used for quality control purposes. MIC data were interpreted in a species-specific manner as suggested [20].

### *ERG11* sequencing

*Candida tropicalis* isolates showing fluconazole resistance were subjected to *ERG11* sequencing using a defined protocol [21]. The genome of *C. tropicalis* MYA-3404 (AAFN00000000.2) was considered the reference wild-type [22]. *ERG11* sequences were analysed and curated by SeqMan Pro software (DNASTAR, Madison, WI, USA) and aligned by MEGA software v7.0 [23] in the presence of the wild-type sequence (AAFN00000000.2) (sequences available at the end of the Supplementary files).

### Multilocus microsatellite typing

Environmental and blood *C. parapsilosis* [24] and *C. glabrata* [25] isolates and all blood isolates of *C. tropicalis* [26] were subjected to respective multilocus microsatellite typing techniques using published methods [24–26]. Different genotypes were defined when two given strains differed in more than one microsatellite marker tested [24–26]. Microsatellite data were analyzed using Bionumerics software v7.6 (Applied Math, Sint-Martens-Latem, Belgium) and dendrograms were constructed using the unweighted-pair group method by average linkages. Microsatellite data were considered categorical values.

### Statistical analysis

Data included in this study were analyzed using SPSS software v27 (PSS Inc. Chicago, IL, USA).

### Availability of sequence data

ITS sequences of *Aureobasidium melanogemum, Naganishia albidus*, and *Naganishia liquefaciens* (MN717161-MN717166) and the *ERG11* sequences obtained for FLZR *C. tropicalis* isolates (MN723553-MN723558) were deposited in GenBank (https://www.ncbi.nlm.nih.gov/genbank/).

## Results

### Patient characteristics

In total, 66 yeast isolates were isolated from blood samples of 51 patients (male (28/51; 54.9%), female (19/51; 37.2%) (no data for four patients). Adults (26/51; 51%) and children (23/51; 45.1%) almost equally acquired candidemia (no data for two patients). The vast majority of the patients were hospitalized in Mustapha Pacha (*n* = 38/51; 74.5%), followed by Beni Messous (*n* = 4; 7.8%) and Tizi Ouzou (each *n* = 4; 7.8%), Parnet (*n* = 2; 3.9%), and Blida, EPH Médéa, and EPH Zemirli (each *n* = 1; 1.96%). Patients were admitted mainly to pediatric (18/51; 35.3%) and ICU wards (15/51; 29.4%), followed by neurology (5/51; 9.8%), gastroenterology (3/51; 5.8%), and other wards (*n =* 10/51; 19.6). Neutropenia (*n* = 9/51; 17.6%), leukemia (*n* = 8/51; 15.7%), abdominal surgery and cancer (each *n* = 4/51; 7.8%) were the most prevalent underlying conditions. Antifungal treatment data were available for only 33 patients (no data for 18 patients), among whom FLZ (*n* = 12/51; 23.5%) and caspofungin (*n* = 7/51; 13.7%) were the most widely used systemic antifungals, followed by AMB (*n* = 3/51; 5.6%) (some patients were treated with more than one antifungal); 41% of the patients (*n* = 21/51) did not receive any antifungals. The mortality rate was 68.6% (*n* = 35) (no data for three patients). The overall mortality rate was 66.6% (35/51), and per species, patients infected with *C. glabrata* showed the highest mortality rate (5/6; 83.3%), followed by *C. tropicalis* (13/16; 81.2%), *C. parapsilosis* (9/13; 69.2%, no data for one patient)*,* and *C. albicans* (7/11; 63.6%, no data for patient). Additionally, the only patient infected with *C. dubliniensis* died. The rest of the patients infected with rare yeasts all survived (*n* = 3).

### Identification of yeast isolates and species distributions and prevalence

**Fig. 1:**
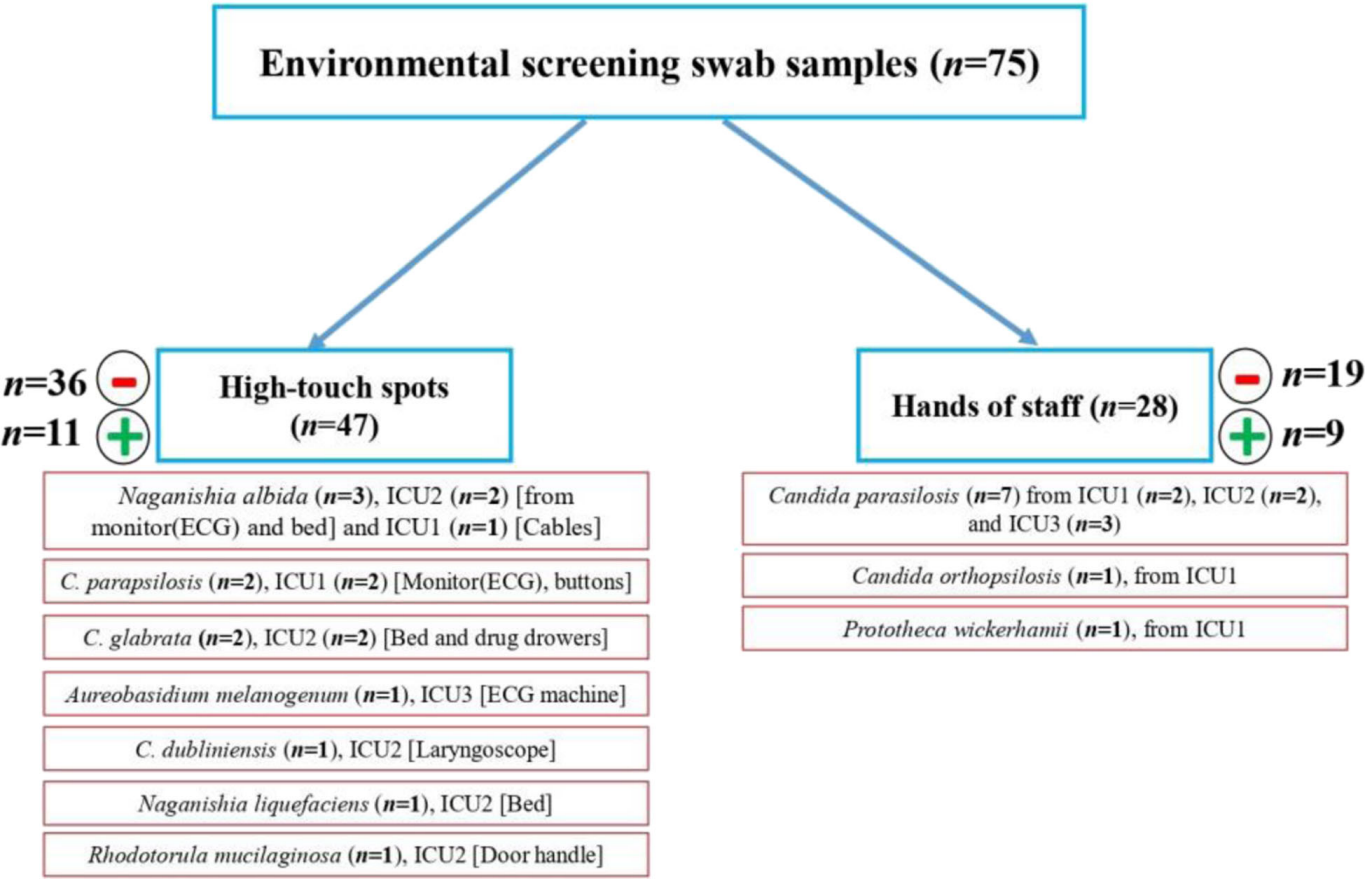
Environmental screening study included 75 swab samples from hands of healthcare workers and high touch areas

*Candida tropicalis* was the most prevalent *s*pecies (16 patients, 19 isolates), followed by *C. parapsilosis* (14 patients, 18 isolates), *C. albicans* (12 patients, 18 isolates), *C. glabrata* (6 patients, 7 isolates), *Clavispora lusitaniae* (*n* = 1), *Meyerozyma elongisporous* (*n* = 1), and *Aureobasidium melanogenum* (*n* = 1) (Supplementary Table 1). Multiple isolates of the same species were recovered from nine patients as follows: *C. albicans* (*n* = 11 from four patients), *C. parapsilosis* (*n* = 6 from two patients), *C. tropicalis* (*n* = 5 from two patients), and *C. glabrata* (*n* = 2 from one patient). Almost one third of the hands of HCWs (9/28) were positive for yeasts, among which 77.7% were *C. parapsilosis* (*n* = 7), followed by *C. orthopsilosis* and *Prototheca wickerhamii* (one isolate each) (Fig. 1, Supplementary Table 1). Approximately 24% of the high-touch areas were positive for yeasts (*n* = 11), including *Naganishia albida* (*Cryptococcus albidus var. albidus*) (*n* = 3; 27.2%) and *C. parapsilosis* and *C. glabrata* (each *n* = 2; 18.1%) (Fig. 1, Supplementary Table 1). Phylogenetic analysis using the neighbor-joining algorithm and 1000 bootstraps was performed to unequivocally identify isolates of *A. melanogenum*, *N. albida*, and *N. liquefaciens* (Supplementary Fig. 1).

### Antifungal susceptibility testing

*Candida albicans, C. parapsilosis,* and *C. glabrata* were susceptible to all antifungal drugs tested. *Candida dubliniensis*, *L. elongisporous*, and *Clavispora lusitaniae* showed MIC values lower than ECV toward all antifungal drugs studied (Table 1 and Supplementary Table 2). FLZR was noted for 31.5% of *C. tropicalis* isolates (*n* = 6; MIC≥8 μg/ml), and 50% were cross-resistant to the three azole drugs tested: 83.3% to FLZ and ITZ (*n* = 5; MIC> 0.5 μg/ml), and 66.6% to FLZ and VRZ (*n* = 4; MIC≥1 μg/ml) (Tables 1 and Supplementary Table 2). Exploring the medical histories of patients infected with fluconazole-resistant (FLZR) isolates showed that three patients received fluconazole (no data for one patient), while one of them did not receive any antifungals in general or azoles in particular during his hospitalization. Isolates from the hands of HCWs were all susceptible to all antifungals tested (Supplementary Table 2). Yeasts isolated from the high touch areas, *N. albida* (*n* = 2)*, N. liquefaciens* (*n* = 1)*,* and *Rhodotorula mucilaginosa* (*n* = 1), showed elevated MIC values for fluconazole (4–64 μg/ml), MFG (8 μg/ml), and AND (8 μg/ml) (Supplementary Table 2).

**Table 1:** Classification of the minimum inhibitory concentration of blood isolates identified in this study based on epidemiological cut-off values and clinical breakpoints

Species	Susceptibility	MIC values (μg/ml)					
		FLZ	VRZ	ITZ	AMB	MFG	ANF
***Candida tropicalis*(*n*** **= 19)**	**<ECV**	12	10	13	19	19	19
	**>ECV**	7	9	5	0	0	0
	**S**	13	15	NA	NA	19	19
	**R**	6	4	NA	NA	0	0
***Candida albicans*(*n*** **= 18)**	**<ECV**	17	17	18	18	18	18
	**>ECV**	1	1	0	0	0	0
	**S**	18	18	NA	NA	18	18
	**R**	0	0	NA	NA	0	0
***Candida parapsilosis*(*n*** **= 18)**	**<ECV**	18	18	18	18	18	18
	**>ECV**	0	0	0	0	0	0
	**S**	18	18	NA	NA	18	18
	**R**	0	0	NA	NA	0	0
***Candida glabrata*(*n*** **= 7)**	**<ECV**	7	7	7	7	6	7
	**>ECV**	0	0	0	0	1	0
	**S**	7	NA	NA	NA	7	7
	**R**	0	NA	NA	NA	0	0
***Candida dubliniensis*(*n*** **= 1)**	**<ECV**	1	1	1	1	1	1
	**>ECV**	0	0	0	0	0	0
***Clavispora lusitaniae*(*n*** **= 1)**	**<ECV**	1	1	1	1	1	1
	**>ECV**	0	0	0	0	0	0
***Lodderomyces elongiporous*(*n*** **= 1)**	**NA**	≤0.125	≤0.03	0.03	0.06	≤0.0156	≤0.0156
***Aureobasidium melanogenum*(*n*** **= 1)**	**NA**	16	0.06	0.06	0.125	1	1

*ECV* Epidemiological cut-off value, *R* Resistant, *S* Susceptible, *NA* Not applicable *MIC* Minimum inhibitory concentration

### *ERG11* sequencing

Six *C. tropicalis* blood isolates resistant to FLZ were subjected to *ERG11* sequencing. Isolate #50 did not carry any nonsynonymous mutations in *ERG11*, and the remaining of five isolates (#58, 61–64) carried nonsynonymous mutation of P56S corresponding to the nucleotide mutation C166T. Moreover, isolate #58 carried an extra nonsynonymous mutation, V234F, corresponding to the nucleotide mutation G700T.

### Typing analysis

**Fig. 2:**
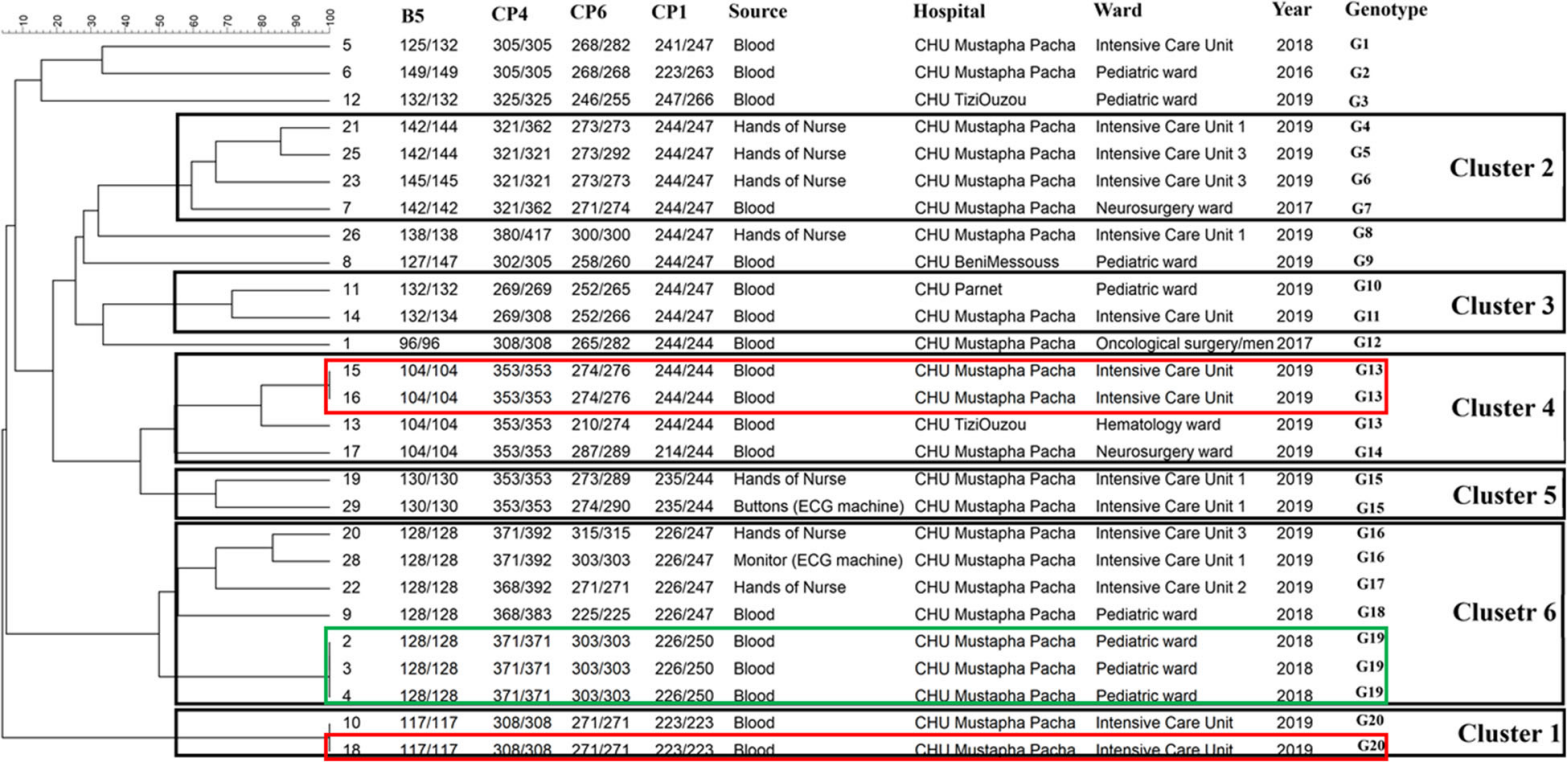
Microsatellite typing of *Candida parapsilosis* isolates recovered from environmental screening and blood samples. Rectangular with the same color contained isolates of the same patients

**Fig. 3:**
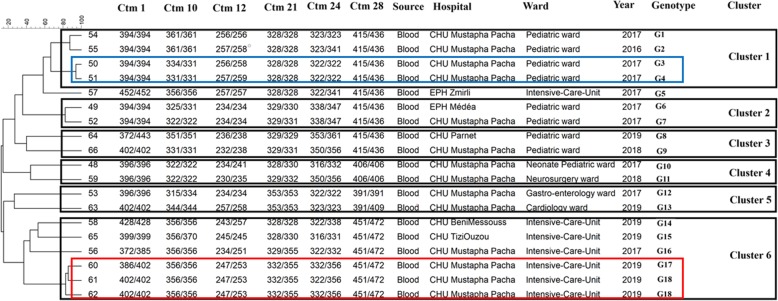
Microsatellite typing of *Candida tropicalis* blood isolates. Rectangular with the same color contained isolates of the same patients

**Fig. 4:**
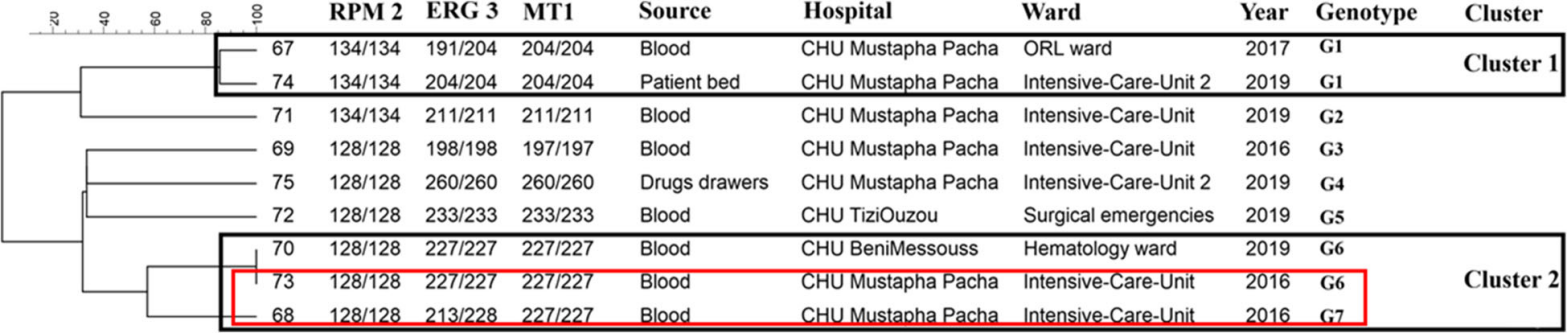
Microsatellite typing of *Candida glabrata* isolates recovered from environmental screening and blood samples. Rectangular with the same color contained isolates of the same patients

*C. parapsilosis* isolates obtained from the hands of HCWs (*n* = 7), ECG monitors and buttons (*n* = 2), and blood (*n* = 18) were subjected to microsatellite typing and revealed 20 genotypes (G1-G20) and six main clusters (C1-C6) (Fig. 2). Among isolates forming defined clusters (*n* = 21; 78%) 61.9% of them (*n* = 13) were identified in intensive care units and 22.7% in pediatric wards (*n* = 5) (Fig. 2). C6 (*n* = 3, hands; *n* = 4, blood) and C2 (*n* = 3, hands; *n* = 1, blood) contained a mixture of blood and hand and/or ECG monitor origins, while those from C4, C3, and C1 were all obtained from blood (Fig. 2). Clonality was observed only for blood isolates collected from Mustapha Pacha hospital (*n* = 7), among which four isolates formed two distinct clones recovered from two patients in 2019 (isolates # 15, 16, and 18 from one patient, and isolate # 10 from another patient, ICU) and the other three isolates (isolates # 2, 3, and 4, pediatric wards) were from another patient in 2018 (Fig. 2). Interestingly, one of the isolates (#13) recovered from a blood sample in TiziOuzou hospital shared the same genotype as those obtained from three other blood samples from Mustapha Pacha hospital (Fig. 2). *Candida tropicalis* isolates formed six clusters representing 18 genotypes and the vast majority of them were obtained from pediatrics (*n* = 8; 42.1%) and ICU wards (*n* = 7; 36.8%) (Fig. 3), among which isolates belonging to C1 (4/4) and C2 (2/2) were from pediatric wards, whereas C6 (6/6) was identified in ICU wards. Clonality was observed only for two FLZR isolates obtained from the same patients (#61 and 62), which were distinct from the first FLZS isolate of the same patient (#60) (Fig. 3). Regarding *Candida glabrata* isolates (7 blood and 2 environmental), 57.1% of the blood isolates (4/7) were recovered from ICU wards (Fig. 4). *Candida glabrata* isolates showed two clusters (C1, *n* = 2; C2, *n* = 3) (Fig. 4). Surprisingly, one of the *C. glabrata* isolates in C1 obtained from a patient bed showed the same genotype as a isolate obtained from a blood sample (Fig. 4). Two patients, one from Mustapha Pacha and one from Beni Messous, were infected with *C. glabrata* isolates that were 100% clonal (#70 and 73) and the two isolates from the same patient (#68 and 73) had the same genotype (Fig. 4).

## Discussion

*Candida tropicalis* with an 81.2% mortality rate showed the highest rate of FLZ resistance, and microsatellite typing highlighted clusters enriched in ICU and pediatric wards. The high prevalence of *C. tropicalis* together with fluconazole resistance is a serious threat hampering the therapeutic efficacy of fluconazole, the frontline antifungal drug used in Algeria. Typing analysis underscored an ongoing *C. parapsilosis* outbreak without an obvious source of infection, as well as inter-hospital transmission of *C. glabrata* and *C. parapsilosis*. A novel amino acid substitution in Erg11p was shown in FLZR *C. tropicalis* isolates.

In concordance with other studies [5, 27], neutropenia, leukemia, and abdominal surgeries were the most prevalent underlying conditions for our patients. The overall crude mortality rate was high (68.6%), and patients infected with *C. glabrata* (83.3%) and *C. tropicalis* (81.2%) showed the highest rates of mortality. Although insertion of central venous catheter and antibiotic treatment are both prominent risk factors for the development of candidemia, these data were scarce and not well recorded in Algerian hospitals. In line with our findings, a candidemia study in South Korea [28] revealed that patients infected with *C. tropicalis* showed the highest mortality rate (44.1%) relative to those infected with other non-*albicans Candida* species. Surprisingly, 44.1% of the patients did not receive any systemic antifungal treatments and among those treated, FLZ was the most commonly used systemic antimycotic. The low price of FLZ and the high cost of echninocandins are among the factors encouraging medical settings of developing and resource-limited countries to use FLZ for the treatment of the vast majority of candidemia cases [5, 11]. We found *C. tropicalis* as the most prevalent *Candida* species, while *C. albicans* ranked third, and *C. parapsilosis* and *C. glabrata* were the second and fourth causes of candidemia in Algeria. The predominance of *C. tropicalis* in Algeria is similar to that in India [5], South Korea [28], and the neighboring country, Tunisia [16]. Moreover, this species is the second cause of candidemia in Brazil [29] and some South East Asian countries [30]. Except for *A. melanogenum* and *C. tropicalis*, which showed elevated MIC values/resistance to azoles, all isolates were susceptible or WT to antifungals tested in this study. The lack of antifungal resistance of *C. glabrata* in this study is similar to that seen in Iranian [11] and Indian studies [31], and in contrast to the relatively high rate of fluconazole and echinocandin resistance in the USA [6]. Surprisingly, 31.5% of the *C. tropicalis* isolates (*n* = 6) were resistant to FLZ, and 50% of those isolates were cross-resistant to the three azoles tested, with 66.6% to VRZ and FLZ, and 83.3% to FLZ and ITZ. Studies in China [32], Taiwan [10], and Denmark [33] observed an alarming increasing trend of azole resistance among *C. tropicalis* isolates. The FLZR *C. tropicalis* isolates were subjected to *ERG11* sequencing, and all but one of the isolates harbored nonsynonymous mutations, among which V234F (G700T) has been previously reported for a FLZR *C. albicans* isolate [34], while P56S (C166T) detected in 83.3% (5/6) of the FLZR isolates was a new mutation. Considering that hydrophobic proline 56 was converted to a polar amino acid of serine (containing a hydroxyl group) and that substitution in neighbor amino acid (A61E) was found solely in FLZR *C. albicans* isolates [35], P56S may cause FLZR. Heterologous expression analysis of both mutations in a wild-type FLZS *C. tropicalis* strain is required to clarify their contribution to azole resistance. The high mortality and high fluconazole resistance rate together with the high prevalence of *C. tropicalis* in Algeria, where candidemic patients are treated mainly by FLZ, pose a serious threat for candidemic patients hospitalized in this country.

To gain insights into infection control measures we conducted a comprehensive environmental screening of high-touch areas and hands of HCWs. *Candida parapsilosis* was the most prevalent yeast species isolated from the hands of HCWs. This result is similar to that in an Italian environmental surveillance study, where *C. parapsilosis* was the most prevalent yeast isolated from HCW hands [36], but in contrast *C. tropicalis* was identified as a major yeast isolated from the hands of Indian HCWs [30, 37]. *Candida parapsilosis* blood and hand isolates belonged to 20 different genotypes, but they formed clusters of genetically similar isolates. Moreover, *C. parapsilosis* isolates obtained from blood samples of two patients were genetically 100% identical. These findings may indicate a hidden source of *C. parapsilosis* that may have started an outbreak in the ICU of Mustapha Pacha hospital, which was not captured by environmental screening, likely due to the low sensitivity of culture [4]. Isolation of two clonal *C. glabrata* blood isolates and two *C. parapsilosis* blood isolates belonging to the same genotype from two hospitals may underscore inter-hospital transmission, likely because some healthcare workers had shifts in both hospitals. Surprisingly, two *C. glabrata* blood isolates recovered from a patient’s bed and blood belonging to the same genotype might be an indication for horizontal transmission of *C. glabrata*, which has been observed in other studies [25]. Although, the lack of isolation of *C. tropicalis* from environmental sources may reject the horizontal transfer of this species in our study, microsatellite typing showed enrichment of genetically similar clusters in ICU and pediatric wards and we could not explain the phenomenon of FLZR acquisition in an azole-naïve patient. We noticed that a primary FLZ-susceptible (FLZS) *C. tropicalis* isolate from a patient was replaced by FLZR isolates during the course of FLZ treatment, likely due to the selective pressure applied by antifungal treatment [38]. Interestingly, the FLZR *C. tropicalis* isolates from that patient shared the same genotype but were different from the FLZS one, which could be explained by microevolution during resistance development [39]. Of note, genotypic variation weas observed for multiple FLZS isolates recovered from the same patient in this study; therefore, genotypic variation may not always be accompanied by resistance development. Interestingly, the isolate of *C. orthopsilosis* from the hands of a HCW may reinforce the hypothesis that, similar to *C. parapsilosis*, it may have been transferred from the hands of HCWs [40]. Moreover, isolation of *A. melanogenum*, *N. albida*, and *N. liquefaciens* from high touch areas, which are reported to have elevated MIC values to various antifungals [41–43] and finding *Aureobasidium melanogenum* in both blood and the environment are worrisome. Findings obtained from environmental screening and microsatellite typing may collectively imply the lack of proper hygiene (both hands and hospital environments) and necessitate the application of effective infection control strategies to eradicate/control fungemia caused by various yeast species. These infection control practices include proper hand hygiene, regular disinfection of hospital environments and high-touch areas, and environmental screening followed by application of typing techniques to identify the source of infection.

The limitations of our study were the retrospective nature of the analysis followed by the lack of additional detailed clinical data and the relatively low numbers of isolates investigated, which is due to underestimation of fungal-related infections in Algeria.

## Conclusion

This study explored the epidemiology of candidemia and the relevant clinical profiles of infected patients in Algeria, for which such data are scant. Moreover, we showed a lack of infection control strategies and antifungal stewardship that should be implemented to improve the patient’s’ outcomes.

## Supplementary information

**Additional file 1: Table S1.** Species identification via MALFI-TOF MS, API Auxacolor, and sequencing. Yeast isolates were primarily identified by API Auxacolor and MALDI-TOF MS and the rare yeast isolates were further characterized by sequencing. **Table S2.** MIC values obtained for the yeast isolates evaluated in the current study. **Figure S1.** Phylogenetic tree for *Aureobasidium melanogenum, Naganishia albida*, and *Naganishia liquefaciens* using neighbor joining algorithm and 1000 bootstraps. Bar shows five nucleotide difference in 100 bps.

## Data Availability

All data obtained in this study were presented in the form of tables, figure, and supplementary data. GenBank data obtained for sequencing of genes of interest are included in this study.

## Abbreviations

MALDI-TOF MS: Matrix-assisted laser desorption ionization time-of-flight
ITS rDNA: Internal Transcribed spacer ribosomal DNA
CLSI: Clinical Laboratory Standard Institute
HCW: Healthcare workers
NAC: Non-*albicans Candida*
MIC: Minim inhibitory concentration
AFST: Antifungal susceptibility testing
SCA: Sabouraud chloramphenicol agar
SDA: Sabouraud dextrose agar
CTAB: Cetyltrimethylammonium bromide
FLZ: Fluconazole
VRZ: Voriconazole
ITZ: Itraconazole
AMB: Amphotericin B
AND: Anidulafungin
MFG: Micafungin
ATCC: American Type Culture Collection
ICU: Intensive care unit
FLZR: Fluconazole-resistant
FLZS: Fluconazole-susceptible
C: Cluster
