# Supplementary material for: Candida tropicalis is the most prevalent yeast species causing candidemia in Algeria: the urgent need for antifungal stewardship and infection control measures
Source: Antimicrob Resist Infect Control. 2020 Apr 7;9:50. doi: 10.1186/s13756-020-00710-z (PMC7140370; doi:10.1186/s13756-020-00710-z)
Supplement: Supplementary file 1 — Additional file 1: Table S1. Species identification via MALFI-TOF MS, API Auxacolor, and sequencing. Yeast isolates were primarily identified by API Auxacolor and MALDI-TOF MS and the rare yeast isolates were further characterized by sequencing. Table S2. MIC values obtained for the yeast isolates evaluated in the current study. Figure S1. Phylogenetic tree for Aureobasidium melanogenum, Naganishia albida, and Naganishia liquefaciens using neighbor joining algorithm and 1000 bootstraps. Bar shows five nucleotide difference in 100 bps. [file 13756_2020_710_MOESM1_ESM.docx]

**Table S1.** Species identification via MALFI-TOF MS, API Auxacolor, and sequencing. Yeast isolates were primarily identified by API Auxacolor and MALDI-TOF MS and the rare yeast isolates were further characterized by sequencing.

| **Strains identified with Maldi-TOF** | **Auxacolor** | | | **Misidentified as** | |
| --- | --- | --- | --- | --- | --- |
|  | **Correctly identified** | **Misidentified** | **Not identified** | **Auxacolor** | **MALDI-TOF MS** |
| **Blood isolates (*n*=65)** | | | | | |
| *Candida tropicalis* (*n*=19) | 18 | 0 | 0 |  |  |
| *Candida albicans* (*n*=18) | 18 | 0 | 0 |  |  |
| *Candida parapsilosis* (*n*=18) | 16 | 2 | 0 | *Sacharomyces cerevisiae* and *Candida albicans* |  |
| *Candida glabrata* (*n*=7) | 7 | 0 | 0 |  |  |
| *Candida dubliniensis* (*n*=1) | 1 | 0 | 0 |  |  |
| *Clavispora lusitaniae* (*n*=1) | 1 | 0 | 0 |  |  |
| *Lodderomyces elongisporus* (*n*=1) | 0 | 0 | 1 |  |  |
| *Aureobasidium melanogenum* (*n*=1)**^*^** | 0 | 0 | 1 |  | *Aureobasidium pullulans* |
| **Isolates from high touch areas (*n*=11)** | | | | | |
| *Naganishia albida* (*n*=3)**^*^** | 0 | 3 | 0 |  | *Naganishia liquefaciens* (*n*=3) |
| *Candida parapsilosis* (*n*=2) | 2 | 0 | 0 |  |  |
| *Candida glabrata* (*n*=2) | 2 | 0 | 0 |  |  |
| *Candida dubliniensis* (*n*=1) | 1 | 0 | 0 |  |  |
| *Naganishia liquefaciens* (*n*=1)**^*^** | 0 | 0 | 1 |  |  |
| *Rhodotorula mucilaginosa* (*n*=1) | 0 | 0 | 1 |  |  |
| *Aureobasidium melanogenum* (*n*=1)**^*^** | 0 | 0 | 1 |  | *Aureobasidium pullulans* |
| **Isolates from hands of staffs (*n*=9)** | | | | | |
| *Candida parapsilosis* (*n*=7) | 7 |  |  |  |  |
| *Candida orthopsilosis* (*n*=1) |  | 1 |  | *Candida parapsilosis* |  |
| *Prototheca wickerhamii* (*n*=1)**^*^** |  |  | 1 |  |  |

* These seven isolates were identified using sequencing targeting internal transcribed sequence (ITS) locus. The ID of the rest of isolates were obtained from MALDI-TOF MS.

**Table S2.** MIC values obtained for the yeast isolates evaluated in the current study.

| **Strain #** | **Species** | **Source** | **MIC values (µg/ml)** | | | | | |
| --- | --- | --- | --- | --- | --- | --- | --- | --- |
|  |  |  | **FLZ** | **VRZ** | **ITZ** | **AMB** | **MFG** | **ANF** |
| **1** | *Candida parapsilosis* | Blood | 0,5 | ≤0,03 | ≤0,03 | 0,25 | 0,5 | 0,25 |
| **2** | *Candida parapsilosis* | Blood | 0,5 | ≤0,03 | ≤0,03 | 0,125 | 0,5 | 0,5 |
| **3** | *Candida parapsilosis* | Blood | 0,5 | ≤0,03 | ≤0,03 | 0,25 | 0,5 | 0,5 |
| **4** | *Candida parapsilosis* | Blood | 0,25 | ≤0,03 | ≤0,03 | 0,06 | 0,5 | 0,5 |
| **5** | *Candida parapsilosis* | Blood | 1 | ≤0,03 | 0,06 | 0,125 | ≤0,0156 | 0,03 |
| **6** | *Candida parapsilosis* | Blood | 0,25 | ≤0,03 | ≤0,03 | 0,25 | 1 | 0,125 |
| **7** | *Candida parapsilosis* | Blood | 0,5 | ≤0,03 | ≤0,03 | 0,25 | 0,5 | 1 |
| **8** | *Candida parapsilosis* | Blood | 0,25 | ≤0,03 | ≤0,03 | 0,5 | 0,5 | 1 |
| **9** | *Candida parapsilosis* | Blood | 1 | ≤0,03 | 0,125 | 0,25 | ≤0,0156 | 0,03 |
| **10** | *Candida parapsilosis* | Blood | 1 | ≤0,03 | 0,06 | 0,06 | 0,5 | 0,25 |
| **11** | *Candida parapsilosis* | Blood | 0,25 | ≤0,03 | ≤0,03 | 0,5 | 1 | 1 |
| **12** | *Candida parapsilosis* | Blood | 0,25 | ≤0,03 | ≤0,03 | 0,25 | 0,5 | 1 |
| **13** | *Candida parapsilosis* | Blood | 1 | ≤0,03 | ≤0,03 | 0,25 | 1 | 2 |
| **14** | *Candida parapsilosis* | Blood | 0,25 | ≤0,03 | ≤0,03 | 0,25 | 0,5 | 0,5 |
| **15** | *Candida parapsilosis* | Blood | 0,5 | ≤0,03 | ≤0,03 | 0,06 | 0,5 | 1 |
| **16** | *Candida parapsilosis* | Blood | 0,5 | ≤0,03 | ≤0,03 | 0,125 | 1 | 1 |
| **17** | *Candida parapsilosis* | Blood | 0,5 | ≤0,03 | ≤0,03 | 0,06 | 0,5 | 0,5 |
| **18** | *Candida parapsilosis* | Blood | 1 | ≤0,03 | ≤0,03 | 0,06 | 0,5 | 0,25 |
| **19** | *Candida parapsilosis* | Hands of Staff | 0,25 | ≤0,03 | ≤0,03 | 0,5 | 0,5 | 1 |
| **20** | *Candida parapsilosis* | Hands of Staff | 0,25 | ≤0,03 | ≤0,03 | 0,06 | 1 | 1 |
| **21** | *Candida parapsilosis* | Hands of Staff | 0,5 | ≤0,03 | 0,06 | 0,25 | 1 | 2 |
| **22** | *Candida parapsilosis* | Hands of Staff | 0,5 | ≤0,03 | ≤0,03 | 0,25 | 1 | 2 |
| **23** | *Candida parapsilosis* | Hands of Staff | 0,25 | ≤0,03 | ≤0,03 | 0,25 | 1 | 2 |
| **25** | *Candida parapsilosis* | Hands of Staff | 0,25 | ≤0,03 | ≤0,03 | 0,125 | 0,5 | 1 |
| **26** | *Candida parapsilosis* | Hands of Staff | 1 | ≤0,03 | ≤0,03 | 0,25 | 0,5 | 1 |
| **28** | *Candida parapsilosis* | Hands of Staff | 0,5 | ≤0,03 | ≤0,03 | 0,25 | 0,5 | 1 |
| **29** | *Candida parapsilosis* | Hands of Staff | 0,25 | ≤0,03 | ≤0,03 | 0,25 | 0,5 | 1 |
| **30** | *Candida albicans* | Blood | ≤0,125 | ≤0,03 | ≤0,03 | 0,25 | ≤0,0156 | ≤0,0156 |
| **31** | *Candida albicans* | Blood | 0,5 | ≤0,03 | ≤0,03 | 0,5 | ≤0,0156 | ≤0,0156 |
| **32** | *Candida albicans* | Blood | ≤0,125 | ≤0,03 | ≤0,03 | 0,25 | ≤0,0156 | ≤0,0156 |
| **33** | *Candida albicans* | Blood | ≤0,125 | ≤0,03 | ≤0,03 | 0,06 | ≤0,0156 | ≤0,0156 |
| **34** | *Candida albicans* | Blood | 0,5 | ≤0,03 | ≤0,03 | 0,25 | 0,06 | ≤0,0156 |
| **35** | *Candida albicans* | Blood | 0,125 | ≤0,03 | ≤0,03 | 0,125 | ≤0,0156 | ≤0,0156 |
| **36** | *Candida albicans* | Blood | ≤0,125 | ≤0,03 | ≤0,03 | 0,125 | ≤0,0156 | ≤0,0156 |
| **37** | *Candida albicans* | Blood | 0,125 | ≤0,03 | ≤0,03 | 0,25 | ≤0,0156 | ≤0,0156 |
| **38** | *Candida albicans* | Blood | ≤0,125 | ≤0,03 | ≤0,03 | 0,25 | 0,06 | ≤0,0156 |
| **39** | *Candida albicans* | Blood | 0,125 | ≤0,03 | ≤0,03 | 0,25 | ≤0,0156 | ≤0,0156 |
| **40** | *Candida albicans* | Blood | ≤0,125 | 0,0156 | ≤0,03 | 0,25 |  | 0,0156 |
| **41** | *Candida albicans* | Blood | ≤0,125 | ≤0,03 | ≤0,03 | 0,5 | ≤0,0156 | ≤0,0156 |
| **42** | *Candida albicans* | Blood | 0,125 | ≤0,03 | ≤0,03 | 0,125 | ≤0,0156 | ≤0,0156 |
| **43** | *Candida albicans* | Blood | 0,125 | ≤0,03 | ≤0,03 | 0,25 | ≤0,0156 | ≤0,0156 |
| **44** | *Candida albicans* | Blood | ≤0,125 | ≤0,03 | ≤0,03 | 0,25 | ≤0,0156 | ≤0,0156 |
| **45** | *Candida albicans* | Blood | 0,25 | ≤0,03 | ≤0,03 | 0,25 | 0,06 | ≤0,0156 |
| **46** | *Candida albicans* | Blood | 2 | 0,06 | 0,125 | 0,25 | ≤0,0156 | 0,06 |
| **47** | *Candida albicans* | Blood | 0,25 | ≤0,03 | ≤0,03 | 0,125 | ≤0,0156 | ≤0,0156 |
| **48** | *Candida tropicalis* | Blood | 0,25 | ≤0,03 | ≤0,03 | 0,25 | 0,03 | 0,03 |
| **49** | *Candida tropicalis* | Blood | 0,25 | ≤0,03 | ≤0,03 | 0,25 | 0,03 | ≤0,0156 |
| **50** | *Candida tropicalis* | Blood | ≥64 | ≥16 | ≥16 | 0,25 | ≤0,0156 | ≤0,0156 |
| **51** | *Candida tropicalis* | Blood | 1 | ≤0,03 | ≤0,03 | 0,125 | ≤0,0156 | ≤0,0156 |
| **52** | *Candida tropicalis* | Blood | ≤0,125 | ≤0,03 | ≤0,03 | 0,25 | 0,03 | 0,03 |
| **53** | *Candida tropicalis* | Blood | 0,5 | 0,125 | ≤0,03 | 0,25 | ≤0,0156 | 0,03 |
| **54** | *Candida tropicalis* | Blood | 2 | 0,125 | 0,25 | 0,125 | 0,03 | ≤0,0156 |
| **55** | *Candida tropicalis* | Blood | 0,5 | ≤0,03 | ≤0,03 | 0,25 | ≤0,0156 | ≤0,0156 |
| **56** | *Candida tropicalis* | Blood | 0,25 | 0,125 | 0,125 | 0,25 | ≤0,0156 | 0,03 |
| **57** | *Candida tropicalis* | Blood | 0,5 | 0,06 | 0,06 | 0,125 | 0,03 | ≤0,0156 |
| **58** | *Candida tropicalis* | Blood | 8 | 1 | 0,5 | 0,5 | ≤0,0156 | ≤0,0156 |
| **59** | *Candida tropicalis* | Blood | 1 | ≤0,03 | ≤0,03 | 0,25 | ≤0,0156 | ≤0,0156 |
| **60** | *Candida tropicalis* | Blood | ≤0,125 | ≤0,03 | 0,5 | 0,125 | ≤0,0156 | ≤0,0156 |
| **61** | *Candida tropicalis* | Blood | >64 | 0,125 | >16 | 0,125 | ≤0,0156 | ≤0,0156 |
| **62** | *Candida tropicalis* | Blood | >64 | 0,125 | >16 | 0,125 | ≤0,0156 | ≤0,0156 |
| **63** | *Candida tropicalis* | Blood | >64 | >16 | >16 | 0,25 | ≤0,0156 | 0,03 |
| **64** | *Candida tropicalis* | Blood | ≥64 | ≥16 | ≥16 | 0,25 | ≤0,0156 | 0,06 |
| **65** | *Candida tropicalis* | Blood | ≤0,125 | ≤0,03 | ≤0,03 | 0,25 | ≤0,0156 | 0,03 |
| **66** | *Candida tropicalis* | Blood | 0,25 | ≤0,03 | ≤0,03 | 0,25 | ≤0,0156 | 0,03 |
| **67** | *Candida glabrata* | Blood | 2 | ≤0,03 | ≤0,03 | 0,5 | ≤0,0156 | 0,03 |
| **68** | *Candida glabrata* | Blood | 1 | ≤0,03 | ≤0,03 | 0,25 | ≤0,0156 | 0,03 |
| **69** | *Candida glabrata* | Blood | 1 | ≤0,03 | 0,06 | 0,25 | ≤0,0156 | 0,03 |
| **70** | *Candida glabrata* | Blood | 2 | ≤0,03 | ≤0,03 | 0,5 | ≤0,0156 | 0,03 |
| **71** | *Candida glabrata* | Blood | 1 | ≤0,03 | ≤0,03 | 0,25 | ≤0,0156 | 0,03 |
| **72** | *Candida glabrata* | Blood | 1 | ≤0,03 | ≤0,03 | 0,25 | ≤0,0156 | ≤0,0156 |
| **73** | *Candida glabrata* | Blood | 1 | ≤0,03 | ≤0,03 | 0,25 | 0,5 | 0,25 |
| **74** | *Candida glabrata* | Patient bed | 2 | ≤0,03 | 0,06 | 0,25 | ≤0,0156 | 0,03 |
| **75** | *Candida glabrata* | Drugs drawer | 1 | ≤0,03 | ≤0,03 | 0,25 | ≤0,0156 | 0,03 |
| **76** | *Candida dubliniensis* | Blood | ≤0,125 | ≤0,03 | ≤0,03 | 0,06 | ≤0,0156 | ≤0,0156 |
| **77** | *Candida lusitaniae* | Blood | 0,5 | ≤0,03 | ≤0,03 | 0,06 | 0,03 | 0,03 |
| **78** | *Lodderomyces elongisporus* | Blood | ≤0,125 | ≤0,03 | 0,03 | 0,06 | ≤0,0156 | ≤0,0156 |
| **79** | *Aureobasidium melanogenum* | Blood | 16 | 0,06 | 0,06 | 0,125 | 1 | 1 |
| **80** | *Prototheca wickerhamii* | Hands of Staff | ND | ND | ND | ND | ND | ND |
| **81** | *Naganishia albida* | Patient bed | 0,5 | 2 | 0,125 | 0,125 | 2 | 4 |
| **82** | *Naganishia albida* | Patient bed | >64 | 1 | 0,5 | 0,25 | >8 | >8 |
| **83** | *Naganishia albida* | Cables | 4 | 0,06 | 0,125 | 0,25 | >8 | >8 |
| **84** | *Candida dubliniensis* | Laryngoscope | ≤0,125 | ≤0,03 | ≤0,03 | 0,125 | ≤0,0156 | ≤0,0156 |
| **85** | *Naganishia liquefaciens* | Patient bed | 64 | 0,25 | 0,25 | 0,5 | >8 | >8 |
| **87** | *Rhodotorula mucilaginosa* | Door handle | 64 | 0,5 | 0,5 | 0,25 | 8 | >8 |
| **89** | *Aureobasidium melanogenum* | ECG machine | 0,25 | ≤0,03 | ≤0,03 | 0,25 | 0,5 | 0,25 |

FLZ; Fluconazole, VRZ; Voriconazole, ITZ; Itraconazole, AMB; Amphotericin B, Micafungin; MFG, ANF; Anidulafungin, ND; Not determined, MIC; Minimum inhibitory concentration

**Figure S1.** Phylogenetic tree for *Aureobasidium melanogenum, Naganishia albida*, and *Naganishia liquefaciens* using neighbor joining algorithm and 1,000 bootstraps. Bar shows five nucleotide difference in 100bps.


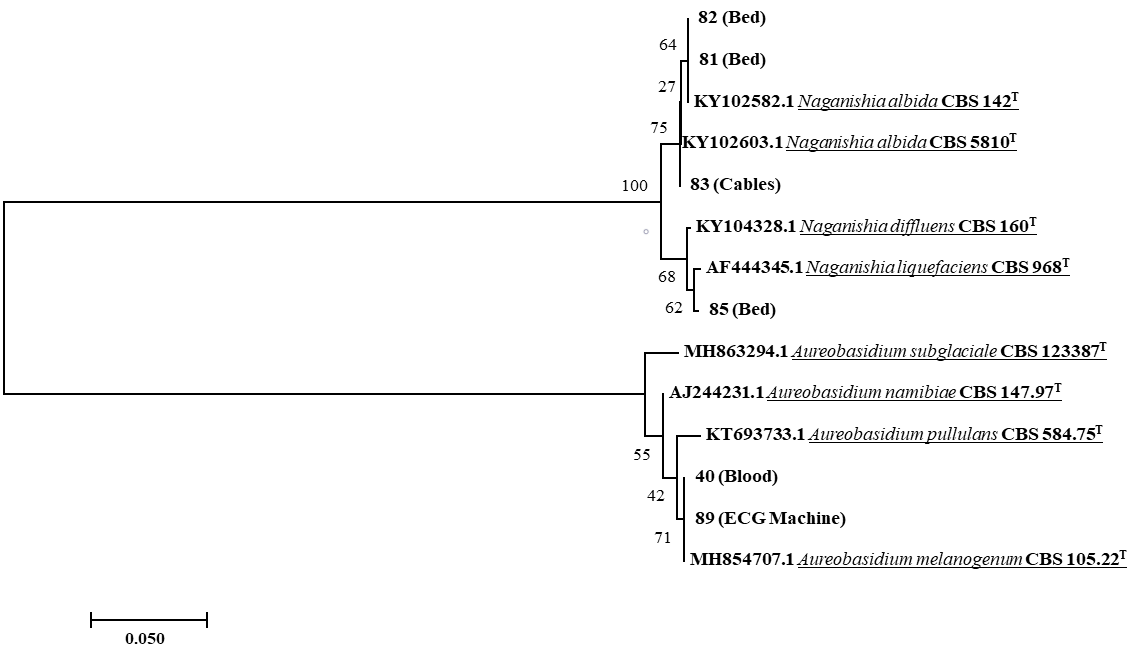


***ERG11*, wild-type sequence**

>ERG11 Candida tropicalis MYA-3404 cytochrome P450 51 (CTRG_05283) partial mRNA

ATGGCTATTGTTGATACTGCCATTGATGGCATCAATTATTTCTTATCCTTATCATTAACTCAACAAATCACCATCTTGGTTGTTTTCCCATTCATCTACAACATAGCATGGCAATTACTTTACTCCTTAAGAAAAGATAGAGTTCCAATGGTTTTCTACTGGATCCCATGGTTTGGTTCTGCTGCTAGTTATGGTATGCAACCATACGAATTCTTTGAAAAGTGCAGATTGAAATATGGTGATGTTTTTTCATTTATGTTATTAGGTAAAGTTATGACTGTTTATTTGGGTCCAAAAGGTCACGAATTCATTTACAATGCTAAATTATCCGATGTTTCTGCTGAAGAAGCTTATACCCATTTGACTACTCCTGTTTTTGGTAAAGGTGTTATTTATGATTGTCCAAACTCTAGATTAATGGAACAAAAGAAGTTTGCTAAATTTGCTTTGACTACTGATTCTTTCAAAACCTATGTTCCAAAGATCAGAGAAGAAGTTTTGAATTATTTTGTTAACGATGTTAGTTTCAAAACCAAGGAAAGAGACCATGGTGTTGCTAGTGTTATGAAAACTCAACCAGAAATCACTATTTTCACTGCTTCTCGTTGTTTATTTGGTGATGAAATGAGAAAGAGTTTCGACAGATCATTTGCTCAATTGTATGCTGACTTGGATAAAGGTTTCACCCCAATCAACTTTGTTTTCCCAAACTTGCCATTACCTCATTACTGGAGACGTGACGCTGCTCAAAGAAAGATATCTGCTCATTACATGAAGGAAATTAAGAGAAGAAGAGAAAGCGGTGATATTGATCCAAAGAGAGATTTGATTGATTCCTTGTTGGTTAACTCTACTTATAAAGATGGTGTTAAAATGACTGATCAAGAAATTGCTAACCTTTTAATTGGTGTTTTGATGGGTGGTCAACATACTTCTGCTTCCACTTCTGCCTGGTTCTTGTTGCATTTGGCTGAACAACCACAATTACAAGATGATCTTTACGAAGAATTGACCAACTTGTTGAAAGAAAAGGGTGGTGACTTGAACGATTTGACTTACGAAGACTTGCAAAAATTACCATTGGTTAACAACACTATTAAAGAAACTTTGAGAATGCACATGCCATTGCATTCTATTTTCAGAAAAGTTATGAACCCATTGAGAGTCCCAAATACCAAATATGTTATTCCAAAAGGTCACTATGTCTTAGTTTCTGCCGGTTATGCTCATACCAGTGATAGATGGTTTGAACACCCAGAACATTTCAACCCAAGAAGATGGGAATCTGATGATACCAAGGCTAGTGCTGTTTCTTTCAATTCTGAAGATACTGTTGATTATGGTTTCGGTAAAATTTCCAAAGGTGTCTCCTCTCCATACTTGCCATTCGGTGGTGGTAGACACAGATGTATTGGTGAACAATTTGCTTATGTTCAATTGGGAACTATTTTGACCACTTATATCTACAACTTCAAATGGAGATTAAACGGTGATAAGGTTCCAGATGTTGATTACCAATCCATGGTTACCTTACCATTAGAACCTGCTGAAATCGTTTGGGAAAAGAGAGATACTTGTATGGTTTAG
